# Analysis of Influence of Experienced Stress and Emotional Eating on Body Mass in a Population of Polish Female Adolescents: PLACE-19 Study

**DOI:** 10.3390/nu18010085

**Published:** 2025-12-26

**Authors:** Dominika Głąbska, Dominika Skolmowska, Dominika Guzek

**Affiliations:** 1 Department of Dietetics, Institute of Human Nutrition Sciences, Warsaw University of Life Sciences (SGGW-WULS), 159C Nowoursynowska Street, 02-776 Warsaw, Poland; dominika_glabska@sggw.edu.pl (D.G.); dominika_skolmowska@sggw.edu.pl (D.S.); 2 Department of Food Market and Consumer Research, Institute of Human Nutrition Sciences, Warsaw University of Life Sciences (SGGW-WULS), 159C Nowoursynowska Street, 02-776 Warsaw, Poland

**Keywords:** stress, emotional eating, body mass, adolescents, Adolescent Stress Questionnaire (ASQ), Emotional Eating Subscale (EE-3), Three-Factor Eating Questionnaire (TFEQ), PLACE-19 Study

## Abstract

**Background/Objectives**: Emotional eating is defined as a coping mechanism characterized by food consumption in response to negative emotions, and it typically involves overconsumption and a preference for energy-dense and highly palatable foods. The aim of this study was to analyze the influence of experienced stress and emotional eating on body mass in a population of Polish female adolescents. **Methods**: This study included 816 participants (aged 15–20 years) recruited within the nationwide PLACE-19 Study using random quota sampling of Polish secondary schools, and data were collected by the Computer-Assisted Web Interview (CAWI). Perceived stress was measured with the Adolescent Stress Questionnaire (ASQ), emotional eating was measured with the Emotional Eating Subscale (EE-3) of the Three-Factor Eating Questionnaire (TFEQ), and self-reported body mass was also recorded. Body mass was interpreted either using standard BMI values for adults or growth reference values for minors. **Results**: Adolescents with excessive body weight reported higher levels of stress on the peer pressure (*p* = 0.0011 for continuous variables; *p* = 0.0016 for categories) and financial pressure component scales (*p* = 0.0319 for continuous variables) than their normal-weight and underweight counterparts. They also displayed higher emotional eating scores across all subscales and for the total emotional eating score (*p* < 0.05 for continuous variables), particularly for anxiety (*p* = 0.0345 for categories). The association was confirmed within mediation analysis, as the direct influence explained 79% of the influence of stress on body mass, and the indirect influence, mediated by emotional eating, explained 21% of the influence of stress on body mass. **Conclusions**: Adolescents with excessive body weight are more prone to stress and emotional eating. The stress itself affects body weight not only directly, but also by affecting emotional eating; therefore, adolescent girls should be taught how to cope with negative emotions using strategies other than increasing food consumption in response to negative emotions. Further studies should assess the mediating role of emotional eating among adolescent girls and evaluate the impact of stress management interventions on body weight.

## 1. Introduction

According to the World Health Organization (WHO), stress is defined as an individually experienced worry or mental tension resulting from a situation perceived as difficult [1]. This global problem is gradually escalating and is currently indicated as one of the health epidemics of the 21st century, which is reflected in rising stress levels; e.g., in the United States of America, its prevalence increased from 10% in 1983 to 30% in 2009 [2]. Before the COVID-19 pandemic, multiple stressors were identified at the individual level (e.g., abuse, educational problems), relationship level (e.g., conflicts with partner, parents, or friends), sociocultural level (e.g., socioeconomic status, norms and values), and community level (e.g., war, neighborhood environment, healthcare access) [3]. The COVID-19 pandemic emerged as a specific and novel stressor, exacerbating previously existing stressors [4].

The umbrella review by Amini-Rarani et al. [5] focusing on the COVID-19 pandemic identified patients, healthcare workers, pregnant women (populations particularly prone to infection or its consequences), and students as the groups experiencing the highest stress levels. This may result from the fact that responses to various stressors in adolescents differ from those in adults, including heightened stress-induced hormonal responses and an increased risk of stress-mediated psychological dysfunction [6]. Similarly, it has been emphasized that the COVID-19 pandemic was a more serious stressor for females, resulting in a higher prevalence of anxiety and depression compared to their male counterparts [7]. Moreover, in an analysis of the psychological and cognitive reactions to the COVID-19-related stress, young females were defined as an especially vulnerable group [8].

The influence of perceived stress on eating behavior may differ, depending on the circumstances, the physiological reaction of the organism, and individual factors [9]. This results from the multidimensional influence of stress on appetite and eating behaviors, a phenomenon observed at both the physiological and psychological levels [10]. Acute stress triggers a ‘fight-or-flight’ reaction, which is associated with an increased heart rate, blood glucose release, and the suppression of non-essential body functions, including hunger [11]. On the other hand, chronic stress is associated with elevated levels of appetite-related hormones, resulting in enhanced food cravings and reward-driven eating behavior [12]. This phenomenon is associated with so-called emotional eating, which is commonly defined as a coping mechanism involving food consumption in response to negative emotions (e.g., stress, upset, furiousness). In such cases, overconsumption is often observed, and energy-dense and palatable food products are chosen [13].

Emotional eating has been identified as an important variable to be taken into account in body mass management, as emphasized in a recent systematic review and meta-analysis by Smith et al. [13]. However, the level of emotional eating may differ depending on the studied group and individual characteristics within that group. In a national study conducted in the United States of America, named the Family Health Habits Survey [14], the groups with the highest emotional eating levels were defined as females, non-Hispanic Caucasians, and younger respondents. The role of the female gender was confirmed in a study of adolescents, also conducted in the United States of America, which indicated that a higher number of stimuli triggers emotional eating in girls than in boys [15]. At the same time, the role of age has been confirmed in several studies, indicating that emotional eating increases from early adolescence [16] and decreases with age in adulthood [17]. Consequently, in the case of adolescent girls, the frequency of emotional eating may reach 43%, compared to 15% for boys in the same population, as indicated for 16-year-olds in the Northern Finland Birth Cohort 1986 (NFBC1986) [18].

Stress-induced body mass changes may include either a decrease or an increase in weight, as indicated in the systematic review by Haidar et al. [19] for a population of students. Moreover, stress-related body mass change may be associated with initial body weight; the Whitehall II Study [20] revealed that for men in the lowest Body Mass Index (BMI) quartile, stress caused body mass reduction, while for those in the highest BMI quartile, it caused weight gain. However, when emotional eating is included as a stress-induced reaction, the typical consequence is weight gain, leading to increased BMI [21] and waist circumference [22]. The role of emotional eating, as a mediator between stress and body mass, has been recently highlighted by several authors, based on studies conducted in populations of children [23], adolescents [24], young adults [25], adults [26], and parents [27].

Little is known about the phenomenon of emotional eating [28], as well as the neural pathways involved, including interactions between neural and neuroendocrine pathways, which are not fully understood [29]. Moreover, a recent review of the current clinical evidence by Dakanalis et al. [30] emphasized that, in spite of the fact that the influence of psychological distress on emotional eating and resultant weight gain is documented, more studies are needed, especially to define the coping mechanisms that are still unknown.

Despite the fact that emotional eating is indicated as an important phenomenon influencing body mass, and stress as a factor commonly activating it, little is still known about the complex influence of experienced stress and emotional eating on body mass. The previous Polish analysis conducted within the PLACE-19 Study (Polish Adolescents’ COVID-19 Experience Study) assessed various psychological aspects of eating behaviors, but so far, it focused on the self-regulation of eating behaviors [31], emotional eating [32], emotional overeating [33], and the association between stress and emotional eating [34]. However, the combined influence of stress and emotional eating on body mass has not yet been addressed. Taking into account that young females are an especially stress-vulnerable group with the highest level of emotional eating, the aim of this study was to analyze the influence of experienced stress and emotional eating on body mass in a population of Polish female adolescents.

## 2. Materials and Methods

### 2.1. General Information

The analysis was conducted within the third phase of the PLACE-19 Study, assessing the psychological aspects of eating behaviors (conducted between 21 January and 17 February 2021). The first phase of the PLACE-19 Study assessed hygienic and personal protective behaviors, while the second phase assessed nutritional behaviors.

The PLACE-19 Study was conducted by the Institute of Human Nutrition Sciences, Warsaw University of Life Sciences (WULS-SGGW). This study was approved by the Ethics Committee of the Central Clinical Hospital of the Ministry of Interior and Administration in Warsaw (No. 2/2021). It was conducted in agreement with the guidelines of the Declaration of Helsinki, and all the participants of this study, as well as their parents/legal guardians, expressed informed consent for participation.

### 2.2. Studied Population of Polish Female Adolescents

Recruitment within the PLACE-19 Study was conducted based on the random quota sampling of Polish secondary schools (separately conducted for each phase of this study). Based on the administrative division of Poland into 16 voivodeships and the division of voivodeships into counties, from each voivodeship, five counties were randomly selected (16 × 5 = 80 counties). Afterwards, based on the national register of the secondary schools, from each county, five secondary schools were randomly selected (80 × 5 = 400 secondary schools). The procedure of gathering a national sample within the secondary schools was chosen due to the high Net Enrollment Rate (NER) in Poland for this level of education (89.38% for December 2019, based on the statistics of the Central Statistical Office (CSO) in Poland [35]). As a consequence, the population of adolescents aged 15–20 years was targeted (typical age for secondary school students in Poland).

The principal of each selected secondary school was contacted and informed about this study. If the principal agreed to the participation of his school in this study, the students, as well as parents/legal guardians (in case of minor participants), were informed about this study, and their informed consents were gathered. The students, providing their informed consent (and the informed consent of their parents/legal guardians), received an electronic link to the study questionnaire to gather the necessary data within the Computer-Assisted Web Interview (CAWI).

The following inclusion criteria were applied: female individuals; age 15–20 years; being a student of a secondary school randomly chosen within a procedure of random quota sampling; informed consent provided; informed consent of parents/legal guardians provided by minor participants. The following exclusion criteria were applied: participation within any previous phase of the PLACE-19 Study; any missing/unreliable data within the questionnaires used for the analysis. The final sample for the conducted study was 816 female participants meeting the inclusion criteria and not excluded due to the exclusion criteria.

### 2.3. Questionnaire Applied Within This Study

To assess the level of perceived stress, the Adolescent Stress Questionnaire (ASQ) [36] was applied and to assess the emotional eating, the Emotional Eating Subscale (EE-3) [37] of the Three-Factor Eating Questionnaire (TFEQ) [38] was applied.

The ASQ was developed and validated by Byrne et al. [36]. Afterwards, it was used within large-scale studies, e.g., the international HELENA Study [39], and other studies in various countries—either in a full or in a shortened version [40,41,42], as it is stated to be the primary questionnaire of choice when measuring adolescent stress [43].

In the conducted study, ASQ was used in its full version, including 58 questions about stressors (including events, emotions, processes) relevant for adolescents. The 58 items are grouped within 10 stress component scales, based on the specific situations (contexts of stressors) associated with (1) home life, (2) school performance, (3) school attendance, (4) romantic relationships, (5) peer pressure, (6) teacher interaction, (7) future uncertainty, (8) school/leisure conflict, (9) financial pressure, and (10) emerging adult responsibility. For each question, a respondent is asked about the previous year and should define how stressful the potential stressor was, while using a 5-point Likert scale, using the categories as follows: ‘not stressful at all’ (1 point on a Likert scale), ‘a little stressful’ (2 points), ‘moderately stressful’ (3 points), ‘quite stressful’ (4 points), and ‘very stressful’ (5 points) [36]. Taking into account the total number of points (maximum: 58 questions × 5 points = 290 points), the stress level for each individual was defined based on the division of the studied group into quartiles of the stress level into lowest stress level within the studied group (first quartile), medium stress level within the studied group (second quartile and third quartile) and highest stress level within the studied group (fourth quartile).

The TFEQ was developed and validated by Stunkard & Messick [38] to measure cognitive restraint of eating (control over consumed food to influence body mass and/or body image), disinhibition (loss of control over food consumption), and hunger (subjective feeling of hunger and food cravings), based on 51 questions about eating behaviors. The following authors broadened the possibility of interpreting the obtained data, presenting the results and validations not only for the total TFEQ, but also for newly defined sub-scales (revised scales), including the Emotional Eating Subscale (EE-3), based on three questions only [37]. The newly defined sub-scales, including EE-3, were validated and used in various studies [44,45,46], including the study conducted for a Polish population [47].

For each question, the respondent is asked about a specific statement and should define whether it is true or false for them, while the statements for EE-3 are as follows: (1) ‘When I feel anxious, I find myself eating’, (2) ‘When I feel blue, I often overeat’, (3) ‘When I feel lonely, I console myself by eating’. Respondent should assess each sentence while using a 4-point response scale, as follows: ‘definitely true’ (4 points), ‘mostly true’ (3 points), ‘mostly false’ (2 points), and ‘definitely false’ (1 point). Based on the total number of points obtained for the sub-scales, the raw scores for individuals were transformed into a result expressed in percent, based on the following equation: raw score minus lowest possible raw score (namely, 3 for EE-3), divided by possible raw score range (namely, 9 for EE-3) and multiplied by 100 [48]. Taking into account the total number of points (maximum: 100% after recalculation), the emotional eating level for each individual was defined based on the division of the studied group into sub-groups, as follows: emotional eating (recalculated result ≥ 50%), and no emotional eating (recalculated result < 50%).

In order to adapt the tools for use in a Polish population, the applied questionnaires were adopted, according to the WHO recommendations [49]. The procedure of adaptation included: (1) forward translation from English to Polish, (2) backward translation from Polish to English, and (3) final polishing conducted by the panel of experts. Afterwards, the validation of the questionnaires included assessment of their internal consistency.

Except for the questions of the ASQ and EE-3, the study questionnaire included the questions allowing to verify the inclusion and exclusion criteria, as well as the questions about body mass and height (self-reported data).

In order to assess body mass, the BMI was calculated by dividing body mass in kilograms by the square of the body height in meters [50]. Afterwards, adult individuals (age > 18 years) were classified, based on the standard BMI cutoffs, as those with underweight (<18.5 kg/m^2^), normal body weight (18.5–25.0 kg/m^2^), overweight (25.0–30.0 kg/m^2^), and obesity (>30.0 kg/m^2^) [51]. At the same time, for minor individuals, the Polish gender- and age-specific growth reference values were used [52], while for each individual values were calculated using the Polish OLAF growth charts [53], and they were classified based on the standard WHO cutoffs, as those with underweight (<5th percentile), normal body weight (5th–85th percentile), overweight (85th–95th percentile) and obesity (>95th percentile) [54].

In order to assess body mass change, an additional question about the body mass and height one year before was asked, while to interpret existing change, the BMI from one year before was calculated separately and compared with the current value (using either the standard BMI value for adults, or growth reference values for minors).

### 2.4. Statistical Analysis

The normality of the data distribution was verified using two different methods depending on the group size—for large samples (*n* > 100), the Kolmogorov–Smirnov test with Lilliefors correction was applied, and for smaller samples, the Shapiro–Wilk test was applied. To compare subgroups, the *t*-Student test or Mann–Whitney U test were used for continuous variables (based on distribution), or the chi^2^ test was used for categorical variables. The linear regression analysis and mediation analysis were conducted to verify the hypothesis of the mediating role of emotional eating for the influence of stress on body mass. The internal consistency of the ASQ component scales and EE-3 subscale was evaluated using Cronbach’s alpha coefficient, with a value of ≥0.70 considered acceptable [55].

The *p* ≤ 0.05 was considered statistically significant. The statistical analysis was conducted using Statistica 8.0 (Statsoft Inc., Tulsa, OK, USA) and Jamovi (The Jamovi project, version 2.6.44, Sydney, NSW, Australia).

## 3. Results

The general characteristics and body mass measures of the Polish female adolescents in the PLACE-19 Study are presented in Table 1. Because the study population included participants under 18 years of age, as well as individuals up to 20 years old, BMI was reported as BMI values and as BMI percentiles for minors. The majority of adolescents were of normal weight; however, the proportion of participants with excessive weight was higher than that of those who were underweight. The distribution of recent body weight change was diverse, with similar proportions of participants reporting weight loss and weight gain, but the majority declaring no recent change in body mass.

Stress scores of the Polish female adolescents in the PLACE-19 Study, assessed with the ASQ, are presented in Table 2. The highest stress burden was observed for home life and school performance component scales, while the lowest levels were reported for school attendance and emerging adult responsibilities. The Cronbach’s alpha values exceeded 0.80 for all domains, indicating good internal reliability, while, for school performance, peer pressure, future uncertainty, school/leisure conflict, and emerging adult responsibility component scales, values exceeded 0.90, indicating very good internal reliability.

Emotional eating scores of the Polish female adolescents in the PLACE-19 Study, assessed with the EE-3 of the TFEQ, are presented in Table 3. Emotional eating was most pronounced in response to loneliness, followed by depressive symptoms and anxiety. The Cronbach’s alpha value for the EE-3 exceeded 0.84, indicating good internal consistency.

Associations between stress continuous variable scores and body mass categories of the Polish female adolescents in the PLACE-19 Study are presented in Table 4. Most stress domains did not differ significantly between underweight, normal body weight, and excessive body weight subgroups. However, adolescents with excessive body weight reported significantly higher scores for peer pressure (*p* = 0.0011) and financial pressure (*p* = 0.0319) than adolescents with normal weight. This suggests that, while overall stress may not be related to body mass status, specific psychosocial stressors, particularly interpersonal and financial ones, may affect adolescents with excessive body weight more than other adolescents.

Associations between emotional eating continuous variable scores and body mass categories of the Polish female adolescents in the PLACE-19 Study are presented in Table 5. Significant differences between weight groups were found for all emotional eating stimuli and for the total emotional eating score (*p* < 0.05). Adolescents with excessive body weight reported higher emotional eating scores than those with normal weight or underweight. These findings highlight a consistent relationship between excessive body mass and the tendency to eat in response to negative emotional states.

Associations between stress categories and body mass categories of the Polish female adolescents in the PLACE-19 Study are presented in Table 6. For the majority of subscales, the proportions of adolescents classified into low, medium, and high stress categories did not differ between body weight groups. The only exception was the peer pressure category (*p* = 0.0016), where individuals with excessive body weight were more frequently represented in the highest stress category than their normal-weight and underweight counterparts. This pattern underscores the importance of peer-related stress in understanding weight-related psychological vulnerabilities in this population.

Associations between emotional eating categories and body mass categories of the Polish female adolescents in the PLACE-19 Study are presented in Table 7. For the majority of emotional eating stimuli, the proportions of adolescents classified as presenting or not presenting emotional eating did not differ between body weight groups. A significant relationship was found only for anxiety (*p* = 0.0345), with adolescents with excessive body weight more likely to be classified as emotional eaters than their normal-weight and underweight counterparts.

The linear regression analysis to predict body mass of the Polish female adolescents in the PLACE-19 Study, based on stress and emotional eating, is presented in Table 8. Regression analysis showed that stress and emotional eating are positive predictors of body mass, and the model explained 17% of the body mass variance (R^2^ = 0.028; F (2, 768) = 11.1; *p* < 0.001).

**Figure 1:**
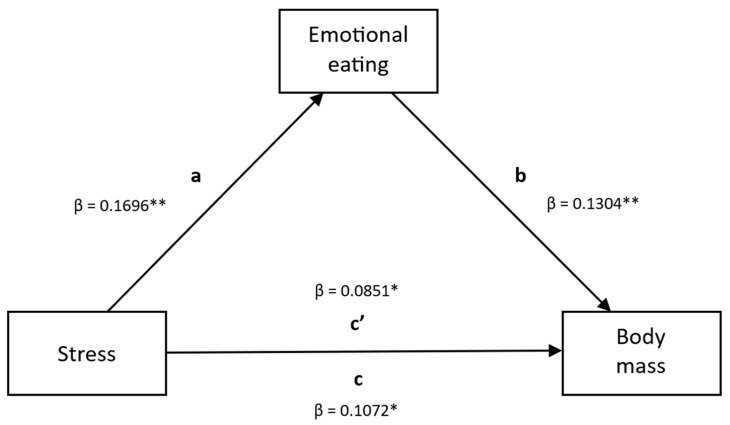
Path model of the mediation analysis for emotional eating and the association between stress and body mass of the Polish minor female adolescents in the PLACE-19 Study. Paths *a* and *b* represent the standardized regression coefficients for the indirect effect of stress on body mass via emotional eating. Path *c* and *c’* are the standardized regression coefficients for the total effect and direct effect, respectively. * *p* < 0.05; ** *p* < 0.001.

The mediation analysis for emotional eating and the association between stress and body mass of the Polish minor female adolescents in the PLACE-19 Study is presented in Table 9 and Figure 1. Stress was found to be a significant positive predictor of emotional eating, and emotional eating was a significant positive predictor of body mass. The total direct and indirect effects were significant, which indicated that emotional eating partially mediates the relationship between stress and body mass among Polish female adolescents, while the direct influence explained 79% of the influence of stress on body mass, and the indirect influence, mediated by emotional eating, explained 21% of the influence of stress on body mass.

## 4. Discussion

The results of this study indicate that, while most adolescents presented a normal body weight, psychological factors, particularly emotional eating, were strongly associated with excessive body mass. Emotional eating emerged as a mediating factor for the association between stress and body mass, in spite of the fact that the direct influence of stress explained 79% of its influence on body mass, and for the indirect influence, mediated by emotional eating, the 21% of the influence of stress on body mass was indicated. These findings suggest that affect-driven eating behaviors, even if they are not the most important, may play a role in shaping weight status among Polish female adolescents.

In their review of emotional eating and weight in adults, Frayn & Knäuper [56] emphasized that emotional eating has a significant negative association with weight loss and should not be ignored when developing weight loss programs for adults. However, adolescence is a critical developmental period marked by increased vulnerability to stress and the maturation of neural systems involved in stress processing [57], meaning that analyses in this age group may yield different results. Indeed, the systematic review by Limbers & Summers [58], which included 13 eligible studies on emotional eating and weight among adolescents, concluded that the available evidence does not consistently support an association between emotional eating and excessive body weight or reduced weight-loss success in this age group. On the other hand, the more recent systematic review and meta-analysis by Demir & Bektaş [59], based on 16 eligible studies, found that emotional eating in adolescents had a positive and medium-sized effect and a significant impact on obesity risk. These findings align with the results obtained in the present study, indicating the most prominent role of stress, but also the mediating role of emotional eating.

This is confirmed by the results of other authors, focusing on stress as a factor contributing to excessive body mass in adolescents, but with improper nutritional habits exacerbating the problem [60]. Moreover, the studies conducted for adolescent girls indicated that the role of stress is observed not only for the body mass during this period, but also 12 months later, which indicates that it is a serious factor potentially associated with early cardiometabolic risk [61].

However, emotional eating behavior, as a moderating factor, must be indicated, as it may contribute to the development of overweightness or obesity, which is emphasized particularly in adolescents [59]. However, in adults, it can be observed not only among individuals with overweight or obesity but also among those with normal body weight or who are underweight [62]. Although emotional eating is most commonly defined as a coping mechanism associated with consumption in response to negative emotions [13], some researchers suggest that emotional eating may also be triggered by positive emotions. For example, underweight adults have been shown to eat less during negative emotional states but more during positive emotional states [62].

Emotional eating, defined as eating in response to emotions rather than hunger, is often associated with higher levels of perceived stress. This was confirmed in the study by Bell et al. [24], who found that emotional eating partially mediated the relationship between perceived stress and eating behavior in adolescents. However, not all adolescents respond to stress with changes in eating behavior. In the study by Sato et al. [63], the authors found that neither chronic stress nor subjective or objective stress reactivity was associated with emotional eating following stress induction. Moreover, it is worth emphasizing that specific stressors, such as peer pressure, appearance concerns, and social relationships, may trigger various eating-related behaviors. The study by Cohrdes et al. [64] revealed that weight- and appearance-related discrimination was associated with a higher risk of eating disorder symptoms in adolescents, whereas interpersonal pressure to be thin and criticism about appearance were associated with increases in disordered eating over time [65].

Moreover, the emotional coping strategies of adolescent girls are still underdeveloped, potentially leading to compensatory eating behaviors. As demonstrated by Lu et al. [66], higher levels of emotional suppression may increase the risk of emotional eating. In girls, higher suppression or a lack of cognitive reappraisal was independently associated with greater consumption of energy-dense foods, which may predispose them to weight gain.

This study has several strengths, including a well-defined population of Polish female adolescents and the use of validated questionnaires to assess stress and emotional eating. However, it also has some limitations. First, its cross-sectional design does not allow for conclusions about direct causality between experienced stress, emotional eating, and body mass. Second, both stress and emotional eating were assessed using self-reported questionnaires, which may be associated with subjective responses, and the instruments were not fully validated in the study population. Third, no data were collected on physical activity or diet quality, both of which could influence body mass and eating behaviors; additionally, body mass and height were assessed based on the self-reported data. Finally, these findings highlight the need for longitudinal studies to better understand the dynamics and causal relationships between stress, emotional eating, and body weight.

## 5. Conclusions

Adolescents with excessive body weight are more prone to stress and emotional eating. The stress itself affects body weight not only directly, but also by affecting emotional eating; therefore, adolescent girls should be taught how to cope with negative emotions using strategies other than increasing food consumption in response to negative emotions. Further studies should assess the mediating role of emotional eating among adolescent girls and evaluate the impact of stress management interventions on body weight.

## Figures and Tables

**Table 1:** General characteristics and body mass measures of the Polish female adolescents in the PLACE-19 Study (*n* = 816).

Characteristics		Median ^2^ (IQR)
Age (years)		17 (2)
Body Mass Index (BMI) (kg/m^2^)		20.8 (4.1)
BMI percentile ^1^		56 (54)
		***n* (%)**
Body mass	Underweight	28 (3.4%)
	Normal body weight	619 (75.8%)
	Overweight	106 (13.0%)
	Obesity	63 (7.7%)
Body mass change	Decrease	237 (29.0%)
	No change	357 (43.7%)
	Increase	223 (27.3%)

^1^—reported for minors (*n* = 771); ^2^—non-parametric distribution confirmed by Kolmogorov–Smirnov test with Lilliefors correction; IQR—Interquartile range.

**Table 2:** Stress scores of the Polish female adolescents in the PLACE-19 Study (*n* = 816), assessed with the Adolescent Stress Questionnaire (ASQ).

Stress Component Scales of the ASQ	Details	
	Cronbach’s Alpha	Median ^1^ (IQR)
Home life	0.90	23 (18)
School performance	0.93	21 (13)
School attendance	0.81	6 (4)
Romantic relationships	0.82	8 (8)
Peer pressure	0.92	14 (13)
Teacher interaction	0.87	14 (11)
Future uncertainty	0.91	11 (8)
School/leisure conflict	0.92	15 (11)
Financial pressure	0.90	8 (9)
Emerging adult responsibility	0.92	7 (8)
Total for general stress	-	134 (66)

^1^—non-parametric distribution confirmed by Kolmogorov–Smirnov test with Lilliefors correction; IQR—Interquartile range.

**Table 3:** Emotional eating scores of the Polish female adolescents in the PLACE-19 Study (*n* = 816), assessed with the Emotional Eating Subscale (EE-3) of the Three-Factor Eating Questionnaire (TFEQ).

Emotional Eating Stimuli Within EE-3	Median ^1^ (IQR)
Anxiety	33.3 (50.0)
Depression	33.3 (66.7)
Loneliness	33.3 (66.7)
Total for general emotional eating	33.3 (55.6)

^1^—non-parametric distribution confirmed by Kolmogorov–Smirnov test with Lilliefors correction; IQR— Interquartile range.

**Table 4:** Associations between stress continuous variable scores, assessed with the Adolescent Stress Questionnaire (ASQ), and body mass categories of the Polish female adolescents in the PLACE-19 Study (*n* = 816).

Stress Component Scales of the ASQ		Body Mass Sub-Groups			*p*
	Details	Underweight (*n* = 28)	Normal Body Weight (*n* = 619)	Excessive Body Weight (*n* = 169)	
Home life	Median (IQR)	20.5 ^1^ (19.5)	23 ^2^ (17)	25 ^2^ (18)	0.2650
School performance	Mean ± SD	21.7 ± 8.1	-	-	0.2369
	Median (IQR)	-	20 ^2^ (13)	22 ^2^ (14)	
School attendance	Median (IQR)	6 ^1^ (5)	6 ^2^ (4)	6 ^2^ (5)	0.7659
Romantic relationships	Median (IQR)	9 ^1^ (9)	8 ^2^ (8)	8 ^2^ (8)	0.2212
Peer pressure	Median (IQR)	13 ^1^ (13) ^ab^	14 ^2^ (12) ^a^	19 ^2^ (14) ^b^	0.0011
Teacher interaction	Median (IQR)	14 ^1^ (12)	13 ^2^ (11)	14 ^2^ (10)	0.6303
Future uncertainty	Median (IQR)	11 ^1^ (8.5)	10 ^2^ (8)	11 ^2^ (7)	0.8270
School/leisure conflict	Median (IQR)	15 ^1^ (11)	14 ^2^ (11)	16 ^2^ (11)	0.3433
Financial pressure	Median (IQR)	6.5 ^1^ (6.5) ^ab^	8 ^2^ (8) ^a^	10 ^2^ (9) ^b^	0.0319
Emerging adult responsibility	Median (IQR)	6.5 ^1^ (6.5)	7 ^2^ (8)	7 ^2^ (8)	0.8812
Total for general stress	Median (IQR)	126.5 ^1^ (70.5)	131 ^2^ (65)	142 ^2^ (72)	0.1549

^1^—non-parametric distribution confirmed by the Shapiro–Wilk test; ^2^—non-parametric distribution confirmed by Kolmogorov–Smirnov test with Lilliefors correction; ^a,b^—different letters in rows indicate statistically significant differences; SD—standard deviation; IQR—Interquartile range.

**Table 5:** Associations between emotional eating continuous variable scores, assessed with the Emotional Eating Subscale (EE-3) of the Three-Factor Eating Questionnaire (TFEQ), and body mass categories of the Polish female adolescents in the PLACE-19 Study (*n* = 816).

Emotional Eating Stimuli Within EE-3	Details	Body Mass Sub-Groups			*p*
		Underweight (*n* = 28) ^1^	Normal Body Weight (*n* = 619) ^2^	Excessive Body Weight (*n* = 169) ^2^	
Anxiety	Median (IQR)	0 (16.7) ^a^	33.3 (33.3) ^b^	33.3 (66.7) ^b^	0.0008
Depression	Median (IQR)	0 (33.3) ^a^	33.3 (66.7) ^a^	33.3 (66.7) ^b^	0.0006
Loneliness	Median (IQR)	0 (66.7) ^a^	33.3 (66.7) ^a^	33.3 (66.7) ^b^	0.0199
Total for general emotional eating	Median (IQR)	0 (44.4) ^a^	33.3 (55.6) ^a^	44.4 (55.6) ^b^	0.0004

^1^—non-parametric distribution confirmed by the Shapiro–Wilk test; ^2^—non-parametric distribution confirmed by Kolmogorov–Smirnov test with Lilliefors correction; ^a,b^—different letters in rows indicate statistically significant differences; IQR—Interquartile range.

**Table 6:** Associations between stress categories, assessed with the Adolescent Stress Questionnaire (ASQ), and body mass categories of the Polish female adolescents in the PLACE-19 Study (*n* = 816).

Stress Component Scales of the ASQ	Body Mass Sub-Groups									*p*
	Underweight (*n* = 28)			Normal Body Weight (*n* = 619)			Excessive Body Weight (*n* = 169)			
	Lowest SL	Medium SL	Highest SL	Lowest SL	Medium SL	Highest SL	Lowest SL	Medium SL	Highest SL	
Home life	12 (42.9%)	8 (28.6%)	8 (28.6%)	147 (23.7%)	296 (47.8%)	176 (28.4%)	37 (21.9%)	80 (47.3%)	52 (30.8%)	0.1447
School performance	5 (17.9%)	12 (42.9%)	11 (39.3%)	143 (23.1%)	314 (50.7%)	162 (26.2%)	30 (17.8%)	81 (47.9%)	58 (34.3%)	0.1472
School attendance	8 (28.6%)	11 (39.3%)	9 (32.1%)	139 (22.5%)	331 (53.5%)	149 (24.1%)	40 (23.7%)	78 (46.2%)	51 (30.2%)	0.2750
Romantic relationships	5 (17.9%)	13 (46.4%)	10 (35.7%)	188 (30.4%)	269 (43.5%)	162 (26.2%)	52 (30.8%)	74 (43.8%)	43 (25.4%)	0.6475
Peer pressure	4 (14.3%)	14 (50%)	10 (35.7%)	129 (20.8%)	328 (53%)	162 (26.2%)	32 (18.9%)	66 (39.1%)	71 (42%)	0.0016
Teacher interaction	5 (17.9%)	15 (53.6%)	8 (28.6%)	145 (23.4%)	306 (49.4%)	168 (27.1%)	29 (17.2%)	88 (52.1%)	52 (30.8%)	0.4812
Future uncertainty	9 (32.1%)	10 (35.7%)	9 (32.1%)	141 (22.8%)	302 (48.8%)	176 (28.4%)	38 (22.5%)	79 (46.7%)	52 (30.8%)	0.6608
School/leisure conflict	5 (17.9%)	15 (53.6%)	8 (28.6%)	133 (21.5%)	325 (52.5%)	161 (26%)	33 (19.5%)	81 (47.9%)	55 (32.5%)	0.5553
Financial pressure	9 (32.1%)	14 (50%)	5 (17.9%)	161 (26%)	287 (46.4%)	171 (27.6%)	32 (18.9%)	81 (47.9%)	56 (33.1%)	0.1881
Emerging adult responsibility	7 (25%)	15 (53.6%)	6 (21.4%)	148 (23.9%)	300 (48.5%)	171 (27.6%)	41 (24.3%)	76 (45%)	52 (30.8%)	0.8314
Total for general stress	7 (25%)	11 (39.3%)	10 (35.7%)	126 (20.4%)	321 (51.9%)	172 (27.8%)	29 (17.2%)	83 (49.1%)	57 (33.7%)	0.4001

SL—stress level—the lowest stress level (first quartile), the medium stress level (the second and third quartiles merged), and the highest stress level (fourth quartile).

**Table 7:** Associations between emotional eating categories, assessed with the Emotional Eating Subscale (EE-3) of the Three-Factor Eating Questionnaire (TFEQ), and body mass categories of the Polish female adolescents in the PLACE-19 Study (*n* = 816).

Emotional Eating Stimuli Within EE-3	Body Mass Sub-Groups						*p*
	Underweight (*n* = 28)		Normal Body Weight (*n* = 619)		Excessive Body Weight (*n* = 169)		
	Emotional Eating	No Emotional Eating	Emotional Eating	No Emotional Eating	Emotional Eating	No Emotional Eating	
Anxiety	4 (14.3%)	24 (85.7%)	146 (23.6%)	473 (76.4%)	54 (32%)	115 (68%)	0.0345
Depression	6 (21.4%)	22 (78.6%)	198 (32%)	421 (68%)	64 (37.9%)	105 (62.1%)	0.1499
Loneliness	8 (28.6%)	20 (71.4%)	253 (40.9%)	366 (59.1%)	78 (46.2%)	91 (53.8%)	0.1708
Total for general emotional eating	6 (21.4%)	22 (78.6%)	189 (30.5%)	430 (69.5%)	65 (38.5%)	104 (61.5%)	0.0707

**Table 8:** Linear regression analysis to predict body mass of the Polish female adolescents in the PLACE-19 Study based on the stress and emotional eating (*n* = 771).

Variable	B	SE B	β	t	*p*	95% CI		Collinearity Statistics	
						LL	UL	Tolerance	VIF
(Intercept)	42.4608	3.4525		12.30	<0.001				
Stress	0.0566	0.0240	0.0851	2.36	0.019	0.0142	0.156	1.03	0.971
Emotional eating	0.1237	0.0343	0.1304	3.61	<0.001	0.0595	0.201	1.03	0.971

SE—standard error; CI—confidence interval; LL—lower level; UL—upper level; VIF—variance inflation factor.

**Table 9:** The mediation analysis for the emotional eating and the association between stress and body mass of the Polish minor female adolescents in the PLACE-19 Study (*n* = 771).

Type	Effect	b	SE	95% CI		β	z	*p*
				LL	UL			
Indirect	Stress ⇒ Emotional eating ⇒ Body mass	0.0147	0.00510	0.00471	0.0247	0.0221	2.88	0.004
Component	Stress ⇒ Emotional eating	0.1189	0.02487	0.0701	0.1676	0.1696	4.78	<0.001
	Emotional eating ⇒ Body mass	0.1237	0.03418	0.0567	0.1907	0.1304	3.62	<0.001
Direct	Stress ⇒ Body mass	0.0566	0.02396	0.0096	0.1035	0.0851	2.36	0.018
Total	Stress ⇒ Body mass	0.0713	0.02382	0.0246	0.1180	0.1072	2.99	0.003

SE—standard error; CI—confidence interval; LL—lower level; UL—upper level.

## Data Availability

Data are provided on request. The data are not publicly available due to ethical restrictions.

## Abbreviations

The following abbreviations are used in this manuscript:

ASQ	Adolescent Stress Questionnaire
BMI	Body Mass Index
CI	Confidence Interval
EE-3	Emotional Eating Subscale
IQR	Interquartile range
LL	Lower Level
SE	Standard Error
SD	Standard Deviation
TFEQ	Three-Factor Eating Questionnaire
UL	Upper Level
VIF	Variance Inflation Factor
WHO	World Health Organization
